
JOURNAL INFORMATION
==============================
NLM Title Abbreviation: Behav Neurol
Journal ID: Behav Neurol
NLM Journal ID: 8914585
Journal ID: BN

Behavioural Neurology

ISSN: 0953-4180
EISSN: 1875-8584
Publisher: Wiley

ARTICLE INFORMATION
==============================
PMCID: PMC5444281
PMID: 19847039
DOI: 10.3233/BEN-2009-0242
Article ID: 520895
Article version: 1
Subjects: Introduction

Introduction

Print publication date: 2009
Electronic publication date: 2009 Oct 21
Volume: 21
Issue: 1-2
First page: 1
Last page: 1
Received 2009 Oct 16; Accepted 2009 Oct 16
Copyright: Copyright © 2009 Hindawi Publishing Corporation and the authors.
Copyright year: 2009
License: This is an open access article distributed under the Creative Commons Attribution License, which permits unrestricted use, distribution, and reproduction in any medium, provided the original work is properly cited.
License URL: https://creativecommons.org/licenses/by/3.0/

==============================
Behavioural Neurology 21 (2009) 1
DOI 10.3233/BEN-2009-0242
IOS Press

### Introduction

Approximately 5.2 million Americans currently hold a diagnosis of AD in the US. Over the next fifty years it is expected that there will be an increase of AD cases from approximately 450,000 to 959,000 new cases per year bringing the prevalence to 16 million cases in the US. This has the potential to more than triple current healthcare costs, with the risk of crippling private and federal healthcare funding across the board. Currently, AD care costs U.S. Medicare and Medicaid over $148 billion dollars per year (from the "2008 Alzheimer's Disease Facts and Figures", by the Alzheimer's Association). With the prevalence of AD doubling with every decade of life after age 75, merely delaying the onset of AD by five years would produce a 50% decrease in the prevalence of disease. Finding a way to prevent Alzheimer's disease will impact millions of people and families that would otherwise suffer from this devastating neurodegenerative process. Not finding a prevention in the next fifty years will have broad catastrophic consequences on U.S. and global healthcare systems. Unlike other diseases such as cardiac disease and cancer that have major impact on today's healthcare, Alzheimer's disease is the only one expected to expand in prevalence at an alarming rate due to increasing longevity worldwide. However, the longer we live healthy and productive lives, the more age related illnesses such as AD will have an impact on our societies.

As Alzheimer's disease pathology appears to begin decades before the average onset of clinical symptoms, prevention therapies must be targeted at pre-clinical stages of disease. In order to do this we must develop biomarkers of underlying disease that accurately pre dict future dementia. Towards a goal of developing pre-clinical markers of AD pathology, we must first understand and develop these biomarkers in clinically relevant disease. Magnetic resonance imaging (MRI) and positron emission tomography (PET) offer great promise as biomarkers for identifying underlying structural, functional and disease specific pathology in AD, MCI, and even preclinical disease processes. Imaging is minimally invasive and provides an opportunity to identify and predict underlying AD in vivo, allowing potential for use as predictive markers, prognostic tools, and research endpoints in development of disease modifying treatment trials.

Work in amyloid PET imaging, fluorodeoxyglucose (FDG) PET, structural and functional MRI imaging are the most active areas today in development of AD biomarkers. In this issue, imaging experts from around the world have contributed their insights by reviewing the latest developments in imaging in the field of Alzheimer's disease research, as well as presenting original work. Collectively, the authors of this issue believe that neuroimaging will play a critical role in discovery of a cure for this pervasive global disease.

Adam S. Fleisher, MD, MAS
*Associate Director of Brain Imaging*
*Banner Alzheimer's Institute*
*Medical Director, Alzheimer's Disease Cooperative Study*
*Assistant Professor, Department of Neurosciences*
*University of California, San Diego, USA*

ISSN 0953-4180/09/$17.00 © 2009 – IOS Press and the authors. All rights reserved
